# ChatGPT improves readability in validated spine patient-reported outcome measures

**DOI:** 10.1016/j.xnsj.2026.100917

**Published:** 2026-06-08

**Authors:** George Abdelmalek, Siraj Shaikh, Daniel Coban, Adam Elkholy, Cyrus Emami, Nikhil Sahai, Ki Hwang, Kumar Sinha

**Affiliations:** a Department of Orthopedic Surgery, St. Joseph’s University Medical Center, Paterson, NJ 07503, United States; b University Spine Center, Wayne, NJ 07470, United States

**Keywords:** ChatGPT, Artificial intelligence, Large language models, Patient-reported outcome measures, Readability, Spine surgery

## Abstract

**Background:**

Spine patient-reported outcome measures (PROMs) frequently exceed recommended health literacy thresholds, limiting accessibility. Large language models (LLMs) such as ChatGPT can simplify medical text, but their effects on validated outcome instruments remain unclear.

**Methods:**

A cross-sectional analysis of validated spine PROMs was conducted. Seventy-seven PROMs identified in a prior readability analysis were revised using ChatGPT 4.0 through a standardized prompt instructing simplification to a sixth-grade reading level. Pre and postrevision readability metrics were assessed using Readable.com across multiple grade-level and linguistic indices. Revised PROMs were additionally evaluated for content fidelity using a predefined taxonomy assessing alterations in response scales, recall timeframes, and item meaning. Differences were analyzed using the Exact Sign Test (α = 0.05).

**Results:**

Eighteen of nineteen linguistic parameters improved significantly following ChatGPT revision (p < .05). Word count decreased by 18%, sentence complexity declined, and all readability indices improved (p < .001). About 7 of 9 grade-level metrics achieved NIH/AMA sixth-grade readability compliance following revision. However, 59.7% of PROMs contained at least one content-related error. The most common errors included alteration of validated response scales (23%), omission or simplification of recall timeframes (18%), and consolidation of multiple items into single prompts (16%).

**Conclusions:**

ChatGPT 4.0 substantially improved the readability of validated spine PROMs but frequently introduced structural modifications affecting validated content. Although LLMs may enhance linguistic accessibility, unsupervised PROM revision risks compromising measurement integrity. Structured implementation strategies incorporating expert review and psychometric validation may be necessary before AI-modified PROMs can be integrated into spine outcomes research.

## Introduction

Patient-reported outcome measures (PROMs) are a core component of spine surgery research and clinical practice. They help capture pain, function, disability, and health-related quality of life from the patient perspective and are widely used across clinical studies, registries, and quality reporting to compare outcomes across diagnoses and procedures [1]. As PROM use expands in value-based care models, this data increasingly informs benchmarking, comparative analyses, clinical decision-making, and reimbursement-linked performance metrics [1].

PROM validity depends on patient comprehension. In the United States, health literacy remains limited for a large proportion of adults. In the 2003 National Assessment of Adult Literacy (NAAL), only 12% of adults demonstrated proficient health literacy [2], and more recent national survey evidence using the Newest Vital Sign similarly suggests that over 60% of U.S. adults have low to moderate health literacy [3]. Accordingly, patient-facing health information is recommended to be written at or below a sixth-grade reading level [4]. When the reading level of a PROM exceeds a patient’s ability, incomplete responses and systematic misunderstanding of item wording, response anchors, or recall timeframes can introduce measurement error and bias [4].

Spine PROMs often exceed recommended readability targets. In a comprehensive assessment by Issa et al. [5] most commonly used spine PROMs were written above recommended levels, and only a minority met stricter thresholds aligned with broad accessibility [5]. Although the readability problem is well described, there is limited evidence evaluating scalable approaches to simplify PROM language while preserving validated content. Because PROMs are commonly self-administered, limited comprehension or misunderstandings can introduce nonrandom measurement error and weaken the validity of data collection and clinical conclusions.

Modifying PROMs presents unique challenges since they are not typical patient education materials. Rather, they are validated measurement instruments with deliberately constructed item wording, response options, recall windows, and domain structure designed to measure specific outcomes (eg, pain severity, functional limitation, disability, and psychosocial health) [6]. The FDA guidance on PROMs emphasizes that content validity and patient comprehension are foundational. It notes that modifying an established instrument may require additional evaluation to ensure it continues to measure the intended concept and performs as initially validated [6]. Accordingly, language simplification is not inherently benign. Even small changes to wording may alter interpretation and compromise comparability between reports.

Large language models (LLMs) such as ChatGPT (OpenAI) offer a scalable approach to text simplification. Prior work suggests LLMs can reduce reading grade level in patient education contexts, but they may also introduce factual or meaning-altering errors [7]. However, PROM revision presents a distinct risk profile because small changes to item meaning, response structure, or recall periods can produce inconsistencies and undermine comparability to validated scoring and historical datasets [6]. To date, readability concerns in spine PROMs have been well documented, but the impact of LLM-driven rewriting on PROM readability alongside content fidelity has not been systematically evaluated [5].

Therefore, the purpose of this study was to evaluate whether ChatGPT 4.0 can improve the readability of validated spine PROMs toward NIH/AMA-recommended sixth-grade standards while preserving content validity. The primary aim was to quantify changes in readability using established indices before and after standardized AI revision. The secondary focus was to assess content fidelity through expert review, focusing on PROM-specific threats to validity such as altered response scales, modified recall timeframes, and changes in item meaning.

## Methods

This study was a cross-sectional analysis that evaluated the effect of ChatGPT on the readability and content fidelity of validated PROMs in spine surgery. All outcome measures were revised using ChatGPT 4.0, after which they were reviewed by fellowship-trained spine surgeons for accuracy.

### PROM selection

Of 77 PROMs related to spine surgery were included in the analysis. These instruments were derived from the cohort evaluated by Issa et al. [5] in a published prior comprehensive readability assessment [5]. The selection of outcome measures represented a range of functional domains, including pain, physical capacity, disability, psychosocial health, and quality of life.

All PROMs were analyzed in their entirety as originally published, including instructions, response scales, and scoring guidelines when applicable. Source documents were obtained in publicly available PDF or image-based formats.

### Revision protocol

Each PROM was revised using ChatGPT version 4.0. A standardized, single-pass prompt was used for all instruments to ensure consistency across revisions. The model was instructed to rewrite each PROM at a sixth-grade reading level in accordance with National Institutes of Health (NIH) and American Medical Association (AMA) readability guidelines. The exact prompt used is as follows:

“Rewrite the following PROM at a sixth-grade reading level consistent with NIH and AMA health literacy recommendations. Preserve the original meaning, structure, response options, and scoring system. Do not alter the number of items or response scales.”

All ChatGPT revisions were performed during a single study interval in February 2024 using ChatGPT 4.0, minimizing the potential effect of model drift during data generation. GPT-4 was publicly available through ChatGPT Plus. Each PROM was revised once during the study period. Because large language models may undergo iterative updates over time, outputs generated after the study interval were not revalidated.

Each PROM was entered in full (including instructions and response anchors) and revised once. No iterative corrections or refinement prompts were used. The AI-generated outputs were compiled into complete postrevision versions for comparative evaluation without any human editing done prior to analysis.

### Readability analysis

pre and postrevision PROMs were analyzed using Readable.com (Readable Ltd.), a validated web-based readability analysis platform. Nine standard grade level readability indices were calculated, including the (1) Flesch–Kincaid Grade Level, (2) Gunning Fog Index, (3) SMOG Index, (4) Coleman–Liau Index, (5) Automated Readability Index, (6) Dale–Chall Grade Level, (7) Spache Grade Level, (8) Fry Graph, and (9) FORCAST Index. These indices were selected because they are commonly used in medical readability research and capture complementary aspects of text complexity, including sentence length, word length, syllable burden, and vocabulary difficulty. They have also been used extensively to evaluate the readability of patient-facing health information and PROMs across orthopaedic and spine literature [5,8,9]. Compliance with NIH/AMA recommendations was defined as achieving a sixth-grade reading level or lower.

### Linguistic parameters

In addition to gradelevel indices, nineteen textual and linguistic characteristics were analyzed and grouped into 2 categories: (1) frequency-based measures normalized per 1,000 words and (2) structural indices normalized by alternative metrics. These parameters were selected based on prior readability analyses of patient education and PROM materials [5,10]. All measures were calculated for each PROM before and after AI revision.

#### Counts per 1,000 words

The following variables were calculated per 1,000 words: letters, syllables, sentences, paragraphs, unique words, Dale–Chall difficult words, Spache difficult words, long words (>12 letters), high-syllable words (>4 syllables), long sentences (>20 syllables), very long sentences (>30 syllables), spelling errors, and grammar errors.

#### Counts per other metrics

Additional structural indices included letters per word, syllables per word, words per sentence, words per paragraph, sentences per paragraph, and word count normalized by pre-ChatGPT word length.

### Content accuracy

Content fidelity was assessed by a fellowship-trained spine surgeon with experience in PROM implementation and spine outcomes research. The reviewer was blinded to readability scores and linguistic parameter results and evaluated each revised PROM against the corresponding original instrument using a predefined error taxonomy. Each PROM was classified by direct comparison of validated structural elements, including (1) preservation of item meaning, (2) construct integrity, (3) response scale validity, (4) recall timeframe, (5) scoring instructions, and (6) domain structure. Because content classification was performed by a single reviewer using a predefined taxonomy, inter-rater reliability was not calculated.

Errors were categorized using a predefined taxonomy that included (1) alteration or removal of validated response scales, (2) combination of multiple items into a single prompt, (3) omission or simplification of key timeframes, (4) change in item focus or meaning, (5) weakening or softening of critical wording, (6) removal of instructions for responding or scoring, (7) reordering of validated items or subscales, (8) omission of critical symptom or activity examples, (9) removal or alteration of sensitive or risk-related items, (10) and oversimplification or distortion of psychosocial constructs.

PROMs were classified as containing ≥1 error or no error. The frequency of each error category was recorded.

### Statistical analysis

Continuous variables were summarized using median and interquartile range (IQR) due to non-normal distribution of readability metrics. pre and postrevision comparisons were conducted using the Exact Sign Test for related samples, due to the variability in the magnitude of change across the PROMs. A 2-tailed alpha of 0.05 was considered statistically significant.

Of 77 paired PROMs were included in all analyses. A priori power analysis determined that the provided sample size exceeded the calculated threshold required to detect a 1 grade level improvement in readability indices. All statistical analyses were performed using SPSS statistical software (IBM Corp).

### Outcomes

The primary outcome of this study was the change in grade level readability relative to NIH/AMA sixth-grade standards. Secondary outcomes included changes in linguistic complexity parameters, total word count, frequency and distribution of content errors, and the proportion of PROMs meeting readability compliance after AI revision.

## Results

A total of 77 PROMs underwent paired pre and post-ChatGPT revision analysis. All instruments were successfully revised and analyzed for readability indices and linguistic parameters. Each PROM served as its own control, and the 77 paired comparisons were evaluated using the Exact Sign Test for related samples.

### Counts per 1,000 words

There were significant improvements observed across nearly all normalized linguistic complexity measures following ChatGPT revision (Table 1). Markers of word-level complexity decreased substantially. Median letters per 1,000 words decreased 45,200 (IQR 44,500–46,000) prerevision to 42,950 (IQR 42,200–43,700) postrevision (median paired difference −225, p < .001). Similarly, syllables per 1,000 words decreased from 15,800 (IQR 15,550–16,050) to 14,380 (IQR 14,100–14,650) postrevision (paired difference −120, p < .001). Lexical difficulty also improved, with reductions in Dale–Chall difficult words (265.5–225.3; −40.2, p < .001), Spache difficult words (110.3–92.5; −17.8, p < .001), long words (>12 letters; 12.5–8.7; −3.8, p < .001), and high-syllable words (>4 syllables; 18.5–10.5; −8.0, p < .001).

**Table 1:** Individual textual parameters in pre- versus post-ChatGPT PROMs (n = 77).

Textual characteristic	Pre-ChatGPT median (IQR)	Post-ChatGPT median (IQR)	Median paired difference (post–pre)	p-value
Letters/1,000 words	45,200 (44,500–46,000)	42,950 (42,200–43,700)	−225	<.001
Syllables/1,000 words	15,800 (15,550–16,050)	14,380 (14,100–14,650)	−120	<.001
Sentences/1,000 words	54.0 (50.0–58.5)	65.3 (62.0–68.7)	+11.3	<.001
Paragraphs/1,000 words	41.2 (38.2–45.0)	31.5 (28.5–34.0)	−10.3	<.001
Unique words	182.7 (175.0–189.5)	195.3 (189.0–202.0)	+12.6	<.001
Dale–Chall difficult words	265.5 (255.0–275.0)	225.3 (215.0–235.0)	−40.2	<.001
Spache difficult words	110.3 (105.0–116.0)	92.5 (85.0–99.0)	−17.8	<.001
Long words (>12 letters)	12.5 (11.0–14.0)	8.7 (7.5–10.0)	−3.8	<.001
High-syllable words (>4)	18.5 (17.0–20.0)	10.5 (9.0–12.0)	−8.0	<.001
Long sentences (>20 syllables)	33.0 (30.5–35.8)	16.2 (14.5–18.5)	−16.8	<.001
Very long sentences (>30 syllables)	9.5 (8.0–11.0)	3.1 (2.5–4.0)	−6.4	<.001
Spelling errors	2.5 (2.0–3.5)	2.4 (2.0–3.2)	−0.1	.200
Grammar errors	3.1 (2.0–4.0)	1.7 (1.2–2.5)	−1.4	.003
Letters per word	4.65 (4.55–4.75)	4.38 (4.30–4.45)	−0.27	<.001
Syllables per word	1.54 (1.50–1.58)	1.42 (1.38–1.46)	−0.12	<.001
Words per sentence	6.2 (5.8–6.7)	8.5 (8.2–8.9)	+2.3	<.001
Words per paragraph	118.5 (112.0–125.0)	140.0 (135.0–148.0)	+21.5	<.001
Sentences per paragraph	3.7 (3.5–4.0)	4.5 (4.2–4.8)	+0.8	<.001
Normalized word count	1.00 (1.00–1.00)	0.82 (0.79–0.86)	−0.18	<.001

Sentence structure shifted toward shorter, more segmented constructions. Sentences per 1,000 words increased from 54.0 (50.0–58.5) to 65.3 (62.0–68.7) (+11.3, p < .001), while long sentences (>20 syllables) decreased from 33.0 (30.5–35.8) to 16.2 (14.5–18.5) (−16.8, p < .001) and very long sentences (>30 syllables) decreased from 9.5 (8.0–11.0) to 3.1 (2.5–4.0) (−6.4, p < .001). Paragraphs per 1,000 words also decreased from 41.2 (38.2–45.0) to 31.5 (28.5–34.0) (−10.3, p < .001).

Spelling errors did not significantly change (2.5 vs. 2.4, p = 0.2). However, grammar errors decreased from 3.1 (2.0–4.0) to 1.7 (1.2–2.5) (−1.4, p = .003).

### Counts per other normalization metrics

Additional structural indices demonstrated consistent simplification following ChatGPT revision (Table 1). Word-level measures declined, with letters per word decreasing from 4.65 (IQR 4.55–4.75) to 4.38 (4.30–4.45) (−0.27, p < .001) and syllables per word decreasing from 1.54 (1.50–1.58) to 1.42 (1.38–1.46) (−0.12, p < .001).

Measures of sentence and paragraph structure also changed significantly. Words per sentence increased from 6.2 (5.8–6.7) to 8.5 (8.2–8.9) (+2.3, p < .001). Words per paragraph increased from 118.5 (112.0–125.0) to 140.0 (135.0–148.0) (+21.5, p < .001), and sentences per paragraph increased from 3.7 (3.5–4.0) to 4.5 (4.2–4.8) (+0.8, p < .001).

Normalized total word count, adjusted relative to pre-ChatGPT word length, decreased from 1.00 to 0.82 (0.79–0.86), corresponding to an 18% reduction in overall length (p < .001).

In total, 18 of 19 evaluated textual parameters demonstrated statistically significant change following ChatGPT revision.

### Grade-level readability

All standard readability metrics demonstrated statistically significant improvement following revision (Figure). The dashed horizontal line represents the NIH/AMA recommended sixth-grade readability threshold. Asterisks denote indices that did not achieve sixth-grade compliance following revision. About 7 of 9 grade level indices met NIH/AMA sixth-grade readability compliance after ChatGPT revision. The 2 indices that did not reach the sixth-grade threshold were the Gunning Fog Index and the FORCAST Index. Across the 7 indices that did achieve compliance, improvements were driven by shorter sentences, reduced complex vocabulary, and decreased word length.

**Figure:**
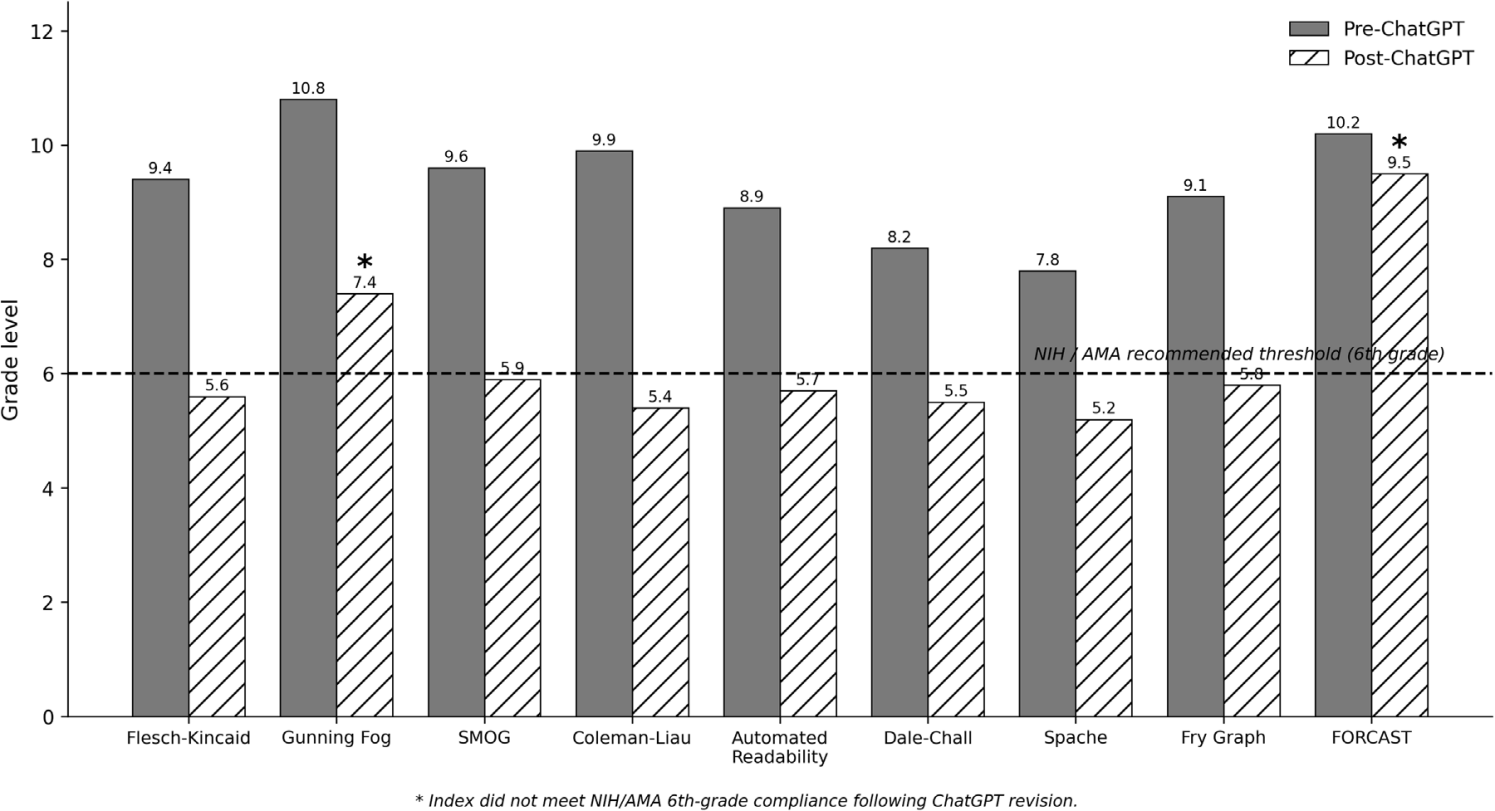
Grade-level readability indices for validated spine PROMs before and after ChatGPT 4.0 revision.

### Content accuracy

Despite improvements in readability, 46 of the 77 PROMs (59.7%) contained at least one content-related error following ChatGPT revision (Table 2). The most common errors involved alteration or removal of validated response scales (23%), omission or simplification of key time frames (18%), and inappropriate combination of multiple items into a single prompt (16%). Changes in item focus or meaning were identified in 13% of PROMs, while removal of response or scoring instructions occurred in 12%. Additional errors included weakening of critical wording (10%), distortion of psychosocial constructs (8%), reordering of validated items or subscales (6%), omission of key symptoms or activity examples (5%), and removal or alteration of sensitive or risk-related items (5%).

**Table 2:** Content fidelity errors in post-ChatGPT PROM revisions (n = 77).

Error type	PROMs affected
Altered/removed validated response scales	18 (23%)
Omitted/simplified key timeframes	14 (18%)
Combined multiple items into 1	12 (16%)
Changed item focus/meaning	10 (13%)
Removed instructions to respond/score	9 (12%)
Weakened/softened wording	8 (10%)
Oversimplified psychosocial constructs	6 (8%)
Reordered validated items/subscales	5 (6%)
Omitted symptom/activity examples	4 (5%)
Removed/altered sensitive items	4 (5%)
≥1 error present	46 (59.7%)

Errors frequently reflected modification of response anchors (such as reduction of multipoint Likert scales), alteration of recall timeframes, or rewording that shifted the intended construct being measured. Representative examples illustrating how ChatGPT revision altered validated wording, response structure, and construct integrity are provided in Table 3.

**Table 3:** Representative examples of pre- and post-ChatGPT revision wording.

PROM (item/element)	Prerevision (validated)	Postrevision (ChatGPT 4.0)	Content-fidelity issue
Oswestry Disability Index (ODI), section 1 (pain intensity), response options	The pain comes and goes and is very mild. The pain is mild and does not vary much. The pain comes and goes and is moderate. The pain is moderate and does not vary much. The pain comes and goes and is very severe. The pain is very severe and does not vary much.	My pain is mild. My pain is moderate. My pain is severe.	Altered/removed validated response scale (Table 2—23%). Six anchors collapsed to 3; the “comes and goes” versus “does not vary” distinction (a validated within-anchor differentiator) was lost.
ODI, instructions (recall window)	Please answer each section, marking only 1 box which best describes your status on average in the past week.	For each question, choose the answer that best describes you. Pick only 1 for each question.	Omitted/simplified key timeframe (Table 2—18%). The “past week” recall window—central to ODI scoring and comparability—was eliminated, and “on average” was dropped.
Neck Disability Index (NDI), separate reading and concentration items	Reading: I can read as much as I want with no pain in my neck. Concentration: I can concentrate fully when I want to with no difficulty.	I can read and concentrate without any neck pain or trouble focusing.	Combined multiple items into 1 (Table 2—16%). Two separate validated NDI items (assessing distinct functional domains) were merged into a single prompt, destroying the per-item scoring structure.
NDI, pain intensity, most severe anchor	The pain is the worst imaginable at the moment.	My pain is very bad right now.	Weakened/softened wording (Table 2—10%). “Worst imaginable” is an anchored extreme designed to capture maximum severity; “very bad” shifts the upper bound of the response distribution
PROMIS pain interference (sample item)	In the past 7 days, how much did pain interfere with your day-to-day activities?	How much does pain stop you from doing your daily activities?	Omitted/simplified key timeframe (Table 2—18%) and altered item focus (Table 2—13%). The 7-day recall was dropped; “interfere with” (the validated construct) was changed to “stop you from,” shifting the item from interference to prevention.

## Discussion

In this cross-sectional analysis of 77 spine surgery patient outcome measures, ChatGPT 4.0 produced consistent and statistically significant reductions in linguistic complexity, including shorter sentences, fewer difficult words, and an 18% reduction in overall word count. The majority of grade level indices met NIH/AMA sixth-grade recommendations following revision. These improvements in readability, however, were accompanied by a substantial rate of content-related errors. Nearly 60% of revised PROMs contained at least one modification affecting validated structure, response scales, recall timeframes, or item meaning. These findings highlight an important tradeoff: efforts to simplify language may inadvertently compromise the measurement integrity.

Unique words per 1,000 words increased modestly following revision despite simultaneous reductions in letters per word, syllables per word, and total word count. This likely reflects restructuring of dense medical phrasing into shorter, more segmented sentence constructions, which increased lexical variation while simplifying overall language complexity [10].

Many studies have demonstrated that commonly used spine surgery PROMs exceed recommended health literacy thresholds, and yet they still play a central role in informed decision-making and guiding outcomes research [1,11]. Given the prevalence of low health literacy among adults in the healthcare system [12], the current complexity of language in these PROMs can greatly limit patient comprehension, introduce response bias, and undermine the reliability of the collected data [13]. The degree of readability improvement observed in this study suggests that LLMs can reduce these linguistic barriers for patients.

These changes likely reflect the underlying training architecture of LLMs, which are optimized to generate conversational text by favoring shorter sentences, common vocabulary, and simple syntax. This likely contributed to the observed reductions in sentence complexity and word length across revised PROMs. Conversely, LLMs do not inherently recognize psychometric constructs or domain-specific scoring frameworks. When prompted to simplify language, the models may prioritize clarity over fidelity, leading to modification of Likert response anchors or the removal of recall periods. This pattern is consistent with prior literature demonstrating that while LLMs perform well in rewriting educational materials, they may alter clinically sensitive content [7,14].

The persistence of the Gunning Fog and FORCAST indices above sixth-grade compliance likely reflects structural limitations of the formulas themselves rather than inadequate simplification. Both indices are heavily weighted toward vocabulary complexity and remain relatively insensitive to sentence shortening when essential medical terminology must be preserved to maintain construct validity. These findings suggest that certain readability formulas may have inherent floor effects when applied to PROM-style instructional text [15,16].

The frequency and nature of content alterations observed are clinically relevant. The alteration of response scales that was noted may affect score distribution and sensitivity of the outcome measures [17,18]. Removal of timeframes may also shift the measurement of certain symptoms from acute to chronic assessment [19]. Consolidation of separate domains will reduce the specificity, and even subtle changes in wording can alter the perceived severity of symptoms. For spine surgeons who rely on PROM data to benchmark outcomes, compare surgical techniques, or support device-related research, even minor structural changes could compromise the comparability of studies across cohorts and timepoints [20]. Unlike with educational materials, PROM revision carries implications beyond readability alone.

There are certain implications of these findings beyond the discourse of AI implementation strategies. This study reinforces readability as a primary barrier to the application of patient-reported measures. Surgeons interpreting PROM data should consider whether patients fully comprehend the instruments used to assess treatment efficacy. At the same time, the results caution against unsupervised AI modification of patient-facing materials. While LLMs may assist with linguistic simplification, their integration into outcomes research must occur within a structured, multistep workflow. A defensible implementation strategy would involve AI-assisted draft revision followed by clinician-led fidelity review, patient comprehension testing, and formal psychometric equivalence analysis before adoption into research or registry settings [6]. This workflow positions LLMs as an accelerant for an initial simplification draft rather than as autonomous editors of validated measurement instruments.

There were several limitations to this study. First, a single LLM version and a single standardized prompt were used. Alternative prompting strategies, iterative refinement loops, or newer model versions may produce different readability-performance and content-fidelity profiles. The analysis was performed in February 2024 using ChatGPT 4.0, and subsequent model versions including GPT-4o (released May 2024) and later iterations were not evaluated. LLM outputs are known to shift across model updates [21], and the present findings should be interpreted as representative of the GPT-4.0 era. Second, content classification was performed by a single fellowship-trained spine surgeon applying a predefined error taxonomy. While this approach provides internally consistent rubric-based scoring, inter-rater reliability was not assessed by design, and future multirater studies are needed to confirm the reproducibility of the error categories reported here. Third, this study evaluated linguistic and structural properties but did not include psychometric testing of the revised PROMs, nor did it directly measure patient comprehension or response behavior. Cognitive interviewing and formal equivalence testing remain necessary steps before any AI-revised PROM could be considered for clinical use.

Future investigations should prospectively evaluate patient comprehension using original, AI-revised, and hybrid AI-expert revised PROMs. Formal equivalence testing with psychometric evaluation is necessary before these modified instruments can be considered for clinical implementation. As artificial intelligence becomes increasingly integrated into healthcare systems, there is a growing opportunity to define standards for responsible AI implementation in outcomes-focused research.

In conclusion, ChatGPT 4.0 significantly improved the readability of spine surgery PROMs but frequently introduced substantial content errors. These findings suggest that while LLMs demonstrate promise as linguistic simplification tools, unsupervised revision risks compromising measurement validity. Structured integration with expert oversight and formal validation will be essential if AI is to be responsibly incorporated into spine outcomes research.

## Author contributions

George Abdelmalek conceived and designed the study. Cyrus Emami and Siraj Shaikh acquired the data. Siraj Shaikh and George Abdelmalek analyzed and interpreted the data. Siraj Shaikh and Adam Elkholy drafted the manuscript. Nikhil Sahai, Kumar Sinha, and Ki Hwang critically revised the manuscript for important intellectual content. All authors approved the final version of the manuscript and agree to be accountable for all aspects of the work.

## Funding

This research received no financial support from any funding agency in the public, commercial, or not-for-profit sectors. This research did not receive any financial support.

## Declaration of competing interests

The authors declare that they have no known competing financial interests or personal relationships that could have appeared to influence the work reported in this paper.
